# Experience in Molecular Genetic Diagnostics of Birt–Hogg–Dubé Syndrome: Characteristics of Identified Mutations and Evolution of the Methodological Approach

**DOI:** 10.3390/ijms27114731

**Published:** 2026-05-25

**Authors:** Irina G. Sermyagina, Dmitry S. Mikhaylenko, Natalya B. Kuryakova, Viktoria V. Musatova, Olga A. Solovova, Anait E. Voskanyan, Ludmila A. Bessonova, Yulia L. Melyanovskaya, Elena I. Kondratyeva, Alexander V. Polyakov, Olga A. Shchagina, Dmitry V. Zaletaev, Sergey I. Kutsev, Vladimir V. Strelnikov

**Affiliations:** 1 Research Centre for Medical Genetics, Moscow 115522, Russia; 2 Institute of Translational Medicine and Biotechnology, I.M. Sechenov First Moscow State Medical University (Sechenov University), Moscow 119991, Russia

**Keywords:** Birt–Hogg–Dubé syndrome, *FLCN* gene, germline variant, sequencing

## Abstract

Birt–Hogg–Dubé syndrome (BHDS) is a hereditary cancer syndrome caused by pathogenic variants in the *FLCN* gene. BHDS is characterized by clinical heterogeneity and similarities with other non-hereditary diseases, which can complicate diagnosis. The aim of our study was to analyze *FLCN* variants in Russian patients and select the optimal diagnostic approach. We studied 121 unrelated patients suspected for BHDS and 29 of their relatives. Germline variants were analyzed using Sanger sequencing and Multiplex Ligation-dependent Probe Amplification (MLPA). Variant annotation was performed according to the ACMG and AMP recommendations. Pathogenic and likely pathogenic (P/LP) *FLCN* variants were identified in 20.7% of patients, including six new variants. The distribution of *FLCN* variants in our cohort was consistent with data obtained from other authors. The mean age of patients with P/LP variants was higher than of those without: 46.91 versus 33.8 years (*p* < 0.05), suggesting the necessity to apply diagnostic criteria in young patients more carefully. The most common clinical manifestation of BHDS was pulmonary cysts/pneumothorax, while the most informative were alterations involving at least two of three organ systems, which was present in all patients with the P/LP variants, but only in 54% without them (*p* = 0.001). BHDS diagnostics involves sequencing exons 4–14 of the *FLCN* gene in patients with proposed clinical criteria. If the result is negative, extensive *FLCN* deletions are excluded using MLPA, and, in the absence of CNV, WGS is performed.

## 1. Introduction

Birt–Hogg–Dubé syndrome (BHDS, OMIM #135150), also known as Hornstein–Knikenberg syndrome, is an autosomal dominant, hereditary cancer syndrome with high penetrance. Clinically, BHDS is characterized by the development of cutaneous fibrofolliculomas, pulmonary cysts, and renal cancer (RC) [1,2]. Fibrofolliculoma are usually multiple, localized on the head, neck, and upper trunk, and represent the most characteristic visible sign of BHDS; however, they may be absent, especially in young patients [3]. Pulmonary cysts are detected in 70–90% of BHDS cases; they are also multiple, most often located in the basal part of the lungs. Over time, the number and diameter of cysts increase and they can cause spontaneous pneumothorax, the main life-threatening complication in BHDS [4,5]. Renal neoplasms develop in 20–30% of patients with BHDS, which may present various tumor types even within a single organ, but chromophobe renal cell carcinoma (ChRCC) or hybrid oncocytic-chromophobe tumors are most commonly identified. In this case, the type of RC helps to suspect BHDS, since ChRCC accounts for less than 5% of all RC cases in adults, but is the most common kidney tumor in patients with BHDS [2,6]. In addition, there are repeated reports of parathyroid carcinoma in patients with BHDS in the absence of pathogenic variants in the *MEN1* gene, which could be associated with multiple endocrine neoplasia syndrome type 1 [7,8].

BHDS is caused by germline inactivating variants in the *FLCN* tumor suppressor gene that encodes folliculin. Various hypotheses exist regarding the molecular pathogenesis of BHDS-associated tumors. For example, according to one hypothesis, FLCN in cooperation with the FNIP2 is involved in the negative regulation of mTOR kinase activity [9]. Another hypothesis supposes that in the absence of FLCN, DNA-dependent protein kinase (DNA-PK) no longer binds the cyclin D1–CDK4/6 complex, resulting in an accumulating the phosphorylated RB1 and, accordingly, the release of the E2F factor, facilitating the G1/S cell cycle checkpoint transition and stimulating cell proliferation [10].

The current relevance of improving the efficiency of molecular genetic diagnostics of BHDS is substantiated by a number of factors. Previously, the frequency of BHDS was roughly estimated at 1 in 200,000 in Caucasians [11]; this frequency is still listed in the Orphanet database (https://www.orpha.net/en/disease/detail/122, access date 19 October 2025). However, recent calculations indicate that the frequency of pathogenic/likely pathogenic (P/LP) germline variants of *FLCN*, for example, according to an analysis of three genomic databases in the United Kingdom, may be in the ranges of 1 in 3000–5000 individuals. Of course, these data should be interpreted considering the incomplete penetrance of BHDS and the patients’ characteristics in this study, and the methods for assessing pathogenicity and reporting variants; but even taking these limitations into account, the frequency of BHDS appears to have been previously significantly underestimated [12]. Professional medical associations issue recommendations for the monitoring and treatment of patients with BHDS. For example, the National Comprehensive Cancer Network (NCCN) guidelines recommend organ-preserving treatment in case of ChRCC in BHDS [13]. However, in cases of multiple renal carcinomas, nephrectomy is necessary [14]. Therefore, the role of proper molecular genetic diagnostics of BHDS is increasing. Here, we summarize the data about analyses of patients with suspected BHDS, performed in the laboratories of the Research Centre for Medical Genetics in 2013–2025 using various methods, and developed a preferred algorithm for genetic testing.

## 2. Results

### 2.1. Localization and Characteristics of the FLCN Gene Variants

**Figure 1:**
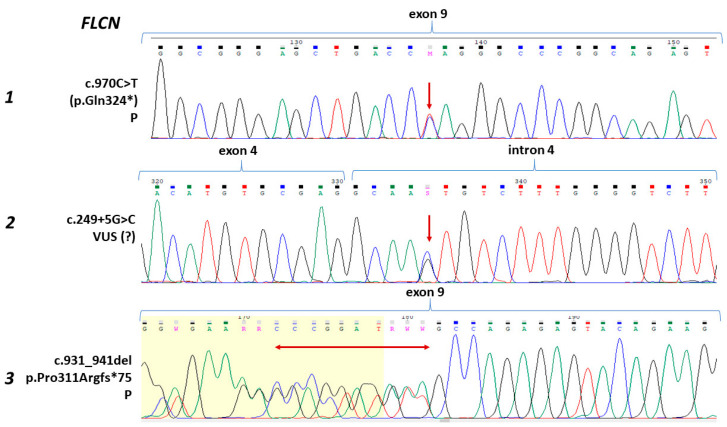
The *FLCN* point variants of various types. Legend: 1–3 are Sanger chromatograms of the samples with variants c.970C>T, c.249+5G>C, and c.931_941del, correspondingly; arrows designate the variant positions; P—pathogenic variant and VUS—variant of uncertain significance.

Sequencing of protein-coding exons 4–14 of the *FLCN* gene was performed in 121 unrelated patients suspected for BHDS (Figure 1). Germline variants were classified by pathogenicity (pathogenic, likely pathogenic, uncertain significance, likely benign, and benign) in accordance with ACMG criteria using combination items in groups PVS1, PS1-4, PM1-5, PP1-5, BA1, BS1-4, BP1-6 [15] and considering a weight point scale from −8 to 8 [16]. Pathogenic, likely pathogenic, and variants of uncertain significance (VUS) were identified in 17, eight, and seven cases, respectively. All identified germline variants were heterozygous.

Among the P/LP variants, various types of inactivating variants (LOF—loss of function) were represented: nonsense variants (8), missense variants (4), frameshift deletions/duplications (11), in-frame deletions (1), and a deletion of an exon part with a splice site (1). Overall, P/LP variants of the *FLCN* were detected in 20.7% (25/121) of patients (Table 1).

The distribution of the *FLCN* variants by alteration type in our patients did not differ from world data (χ^2^, *p* > 0.05) taken from the Franklin and ClinVar databases (accessed 10 November 2025). Previously undescribed variants were detected in six cases: c.214dup, c.1538+5G>C, c.1528G>T, c.390C>G, c.249+5G>C, and c.931_941del. The most common pathogenic variant in our samples was c.1285del, a cytosine deletion in a mutagenesis “hot spot”, the C8 tract in exon 11 of the *FLCN* gene. The second common LP variant c.1300G>C was also localized in exon 11.

Since the implementation of the Multiplex Ligation-dependent Probe Amplification (MLPA) method for determining the *FLCN* gene copy number variation (CNV) into practice at our laboratory, the 26 most recent patients without identified *FLCN* point P/LP variants were analyzed using MLPA. CNV of the *FLCN* were not detected in any of the examined samples.

We classified variants by pathogenicity with the worldwide used ACMG criteria. However, the modern alternative criteria systems exist that could be used to categorize germline variants in the *FLCN* gene. These are the CanVIG-UK Consensus Specification for Cancer Susceptibility Genes of ACGS Best Practice Guidelines for Variant Classification (v3.21) (https://www.cangene-canvaruk.org/canvig-uk-guidance (accessed on 4 May 2026)) and the ACGS Best Practice Guidelines for Variant Classification in Rare Disease 2024 (https://www.genomicseducation.hee.nhs.uk/wp-content/uploads/2024/08/ACGS-2024_UK-practice-guidelines-for-variant-classification.pdf (accessed on 4 May 2026)). These criteria are suitable for germline variant interpretation of hereditary cancer syndromes and rare hereditary diseases, correspondingly, we also provide CanVIG assessment in the Table 1.

The VUSes were examined in more detail (Table 2). These included five missense variants and two variants near canonical splice sites. Variant allele frequency was estimated using the gnomAD v4.1.1 database. To exclude common neutral polymorphisms absent in gnomAD but present in the Russian population, we checked for variants in the RuExAc database up to 8 May 2026. The RuExAc database includes sequencing results for 2910 clinical exomes, performed at the Research Center for Medical Genetics. Three of the seven variants with ultra-low frequency were detected in gnomAD. We have detected the c.346C>A (p.Gln116Lys) heterozygous variant in the RuExAc database with a frequency of approximately 0.1% (threshold for applying the ACMG PM2 criterion for autosomal dominant disease) in three patients with diagnoses other than BHDS.

The missense variants (VUS) were processed by various predictor programs using different methods for predicting variant pathogenicity. For example, the Alpha Missense program (https://alphamissense.hegelab.org/ (accessed on 4 May 2026)) uses protein structure modeling as the main algorithm; PolyPhen-2 (http://genetics.bwh.harvard.edu/pph2/ (accessed on 4 May 2026)) is used for alignment of homologous gene regions; and REVEL (integrated into the Ensembl Genome Browser, http://www.ensembl.org/Homo_sapiens/ (accessed on 4 May 2026)) works with a combination of results from 13 different predictors of known SNPs. We also provided recommended scores for these variants from commonly used genomic annotators: ClinVar (https://www.ncbi.nlm.nih.gov/clinvar/), Varsome (https://varsome.com/), and Franklin (https://franklin.genoox.com/) up to 12 May 2026. The c.346C>A (p.Gln116Lys) variant with a frequency of 0.1% in the RuExAc samples in adult patients without signs of BHDS was characterized as a likely benign (LB) or close to LB variant by predictors. The same result was obtained for the c.247G>A (p.Glu83Lys) substitution regarding the low predicted pathogenicity. In our opinion, c.346C>A and c.247G>A should be considered as LB variants in the absence of clarifying data from segregation analysis and functional studies. In contrast, the variants c.390C>G (p.Ser130Arg), c.715C>T (p.Arg239Cys), and c.1067T>C (p.Leu356Pro) with scores generally greater than 0.9 were characterized as pathogenic changes by predictors and should be reported with a recommendation for their further validation.

VUSes—potential splicing variants—were tested using dbscSNV (a database of potential human SNVs within splicing consensus regions, http://www.liulab.science/dbscsnv.html (accessed on 4 May 2026)), splice site predictor programs SpliceAI (https://spliceailookup.broadinstitute.org/ (accessed on 4 May 2026)), and MaxEntScan (https://genebe.net/tools/maxentscan (accessed on 4 May 2026)). VUSes—missense variants—were also tested by splicing predictors; no new potential aberrant splice sites were detected among them. Variants c.249+5G>C and c.1538+5G>C were characterized by a high score in favor of the loss of donor splice site according to the prediction programs; particularly SpliceAI showed scores > 0.8. It should be noted that ClinVar reported conflicting pathogenicity estimates for VUS/P, while Varsome currently classifies the variant as LP. This is because the variant was reported as pathogenic by colleagues from the Center for Genomic Medicine, Copenhagen University Hospital, in late December 2025 (variation/condition record RCV006274429.1) in addition to the case in our laboratory. Following segregation analysis in the family (see Section 2.3 below), we reclassified c.249+5G>C as LP. Based on the data available as of early 2026, we believe that the c.249+5G>C pathogenicity should be classified no lower than LP.

### 2.2. BHDS Phenotypes in Observed Patients

The clinical data were available for 90.9% (110/121) of patients at the time of diagnosis. Data for 28 from them were restricted by sex, age, preliminary diagnosis of BHDS, and description of the first clinical examination; 82 patients had more comprehensive clinical information. The distribution of patients with P/LP variants of *FLCN* by gender did not differ from the overall cohort (*p* > 0.05). Average age in the group of patients with P/LP variants was 46.91 (95% CI: 40.01–53.81) years and the distribution was symmetrical. Average age of patients without P/LP variants was 29.51 (95% CI: 25.92–33.1) years, the median age was 24 years, and the ratio of the asymmetry coefficient modulus to its standard error was 3.04, which indicates the presence of asymmetry in the distribution. Considering the asymmetry of distribution, we compared the groups using the nonparametric Mann–Whitney test. Average age of patients with P/LP variants was higher than that of patients without (*p* < 0.05). However, the criteria for sample formation were formulated at the time of the start of molecular genetic diagnostics for a wide range of indications, although they were based on the clinical signs of the disease. It is advisable to exclude from the samples those patients in whom BHDS was rejected after differential diagnosis: patients with acquired emphysema and chronic obstructive pulmonary disease, a solitary cyst in the kidney, tuberous sclerosis, chromosomal pathology 46,XX,inv(10)(p.11.2;q21.1), α-1-antitrypsin deficiency with *SERPINA1* P/LP variants, tumor types that are not characteristic after reevaluation for BHDS, such as skin papilloma, as well as patients with strong signs of congenital connective tissue dysplasia (kyphosis, scoliosis, and other skeletal anomalies; flat feet; Marfan-like phenotype; joint hypermobility and habitual dislocations; striae and hyperextensibility of the skin; myopia; mitral valve insufficiency). As a whole, 14 of the 121 potential patients ultimately received alternative diagnoses after genetic testing. After that, the number of patients without P/LP variants was reduced to 82, with an average age of 33.81 ± 1.76 (standard error of the mean) years, and with no significant asymmetry in the distribution, close to normal. Their average age was still different from the group with P/LP variants in the *FLCN* by Mann–Whitney test (*p* < 0.05).

As a whole, 14 cases with P/LP variants and 68 cases without P/LP variants were acceptable for comparison by various BHDS manifestations during their life. We have compared their genotype-phenotype characteristics (Table 3). The most common alteration was lung pathological changes with cysts and/or spontaneous pneumothorax (*p* = 0.013). Despite the differential diagnosis, a significant number of adolescents and young adults with a history of spontaneous pneumothorax remained in the group without P/LP variants. These symptoms allow to recommend BHDS molecular diagnostics, but could also result from other unidentified causes.

Fibrofolliculomas were observed in only eight patients, but with an incidence of 29% vs. 6% (*p* = 0.022, α = 0.05) in the group with P/LP variants vs. non-mutated, correspondingly. This indicates the importance of the most prominent skin BHDS manifestations as a criterion for *FLCN* testing, despite its relatively low frequency. The “renal tumor” feature included not only ChRCC but also clear cell and papillary carcinomas, because they cannot be excluded as part of BHDS. There was one case of clear cell RCC in the group with P/LP variants. In contrast, there were cases of solitary ChRCC and oncocytomas in the group without P/LP variants, which may be sporadic.

The most informative alteration for BHDS was multiple target organ involvement—ChRCC in both kidneys, a combination of pulmonary cysts with recurrent pneumothorax, cutaneous fibrofolliculomas, and/or RC. Complex pathological changes in target organs were present in all patients with P/LP variants, but only in 54% of patients without P/LP variants in the *FLCN* (*p* = 0.001). This fact highlights the importance of a multidisciplinary approach during first medical genetic counseling and recommendation for molecular genetic testing.

### 2.3. Management of P/LP Variant Carriers and Examination of Relatives

**Figure 2:**
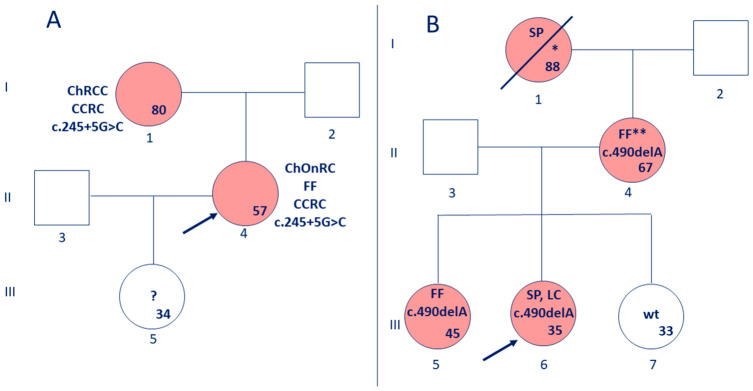
Families with BHDS. Legend: (**A**)—family with affected members harboring the c.245+5G>C and (**B**)—c.490delA variants (red); ChRCC—chromophobe renal cell carcinoma, CCRC—clear cell renal carcinoma, ChOnRC—hybrid chromophobe–oncocytic renal tumor, FF—fibrofolliculomas, SP—spontaneous pneumothorax, LC—lung cysts, wt—no P/LP variant in the *FLCN*, ?—presence of P/LP in the *FLCN* is unknown, *—specimen is unavailable (died before this study), **—MRI of targeted organs is recommended; each family member marked with Arabic numeral, generation—with Roman numeral, the proband is indicated by an arrow.

After identifying *FLCN* germline variants in probands, we examined 29 of their relatives to find asymptomatic carriers of P/LP variants or perform segregation analysis and VUS reclassification in 18 families (Table 4). As a result, ACMG criteria/points were added to pathogenicity of VUS c.249+5G>C (rendering this variant a LP), which was identified in a proband with fibrofolliculomas, primary clear cell carcinoma and hybrid chromophobe–oncocytic renal cell carcinoma. This variant was absent in the gnomAD control samples; the splice site loss score according to the SpliceAI predictor (https://spliceailookup.broadinstitute.org/ (accessed on 4 May 2026)) was 0.88. The variant was inherited by the proband from his mother with a history of ChRCC (Figure 2A). In total, nine asymptomatic siblings and children with the *FLCN* P/LP variants were identified; for example, the patient’s sister with the pathogenic variant c.490delA (Figure 2B). During post-test medical genetic counseling, they were offered a dynamic surveillance according to the European consensus. Notably, it contains recommendations in addition to annual examination of target organs, choosing resection of renal tumors more than 3 cm in diameter, as well as avoiding air travel and physical activity that can cause pneumothorax [17].

## 3. Discussion

Recurrent P/LP variants in our cohort were localized in exon 11 of the *FLCN*. This is consistent with data from other authors that the most common pathogenic variants are localized in exon 11 and in many families are represented by single-nucleotide deletions or duplications with frameshifts in the C8 tract: c.1285del, c.1285dup [2,18,19]. Researchers from China have shown that about 80% of Asian patients with confirmed BHDS carry the P/LP variant in exons 7, 9, 11, 12, and 13 of the *FLCN* gene and suggested starting molecular testing from these loci [18]. Approximately 71% (17/24) of the P/LP variants in our study were localized in these exons, and even a P/LP variants rate of 80% does not seem to provide sufficient clinical sensitivity. In our opinion, at least exons 4–14 of the *FLCN* gene should be routinely sequenced.

The proportion of extended *FLCN* deletions among all other types of LOF variants in patients with BHDS is approximately 4–10%, according to data from other researchers [18,20]. Considering 25 cases with point P/LP variants in 121 probands, the expected number of patients with a large *FLCN* CNV among the 26 tested by the MLPA is less than one, which explains the absence of such cases in our study. Nevertheless, we propose to include MLPA analysis of large germline deletions of the *FLCN* as a second step in the molecular genetic diagnostics of BHDS after excluding point P/LP variants in the coding regions. It allows us to identify the cause of BHDS in some patients and, probably, to describe new pathogenic founder germline variants. For example, an MLPA study of BHDS patients in Anhui province in China demonstrated cases of *FLCN* deletion, which is found in the Feidong district 8.1 times more frequently than in the province as a whole. This pathogenic deletion includes exons 1–3 and their flanking regions [20,21]. The proportion of this deletion in Anhui Province reached 11% of all germline pathogenic variants in the BHDS patients [22]. Also, a study of 278 Swedish patients with suspected BHDS was published that revealed a pathogenic variant c.779+1G>T with a founder effect in the *FLCN* gene in 44 families with a frequency of 57% among all identified P/LP variants [23].

WGS and reclassification of VUS in non-coding regions of the genome is currently a promising area of medical genetics, and successful application of the minigene method has already been described for the *FLCN* gene [24]. WGS in patients with negative routine testing is important for another reason. Due to the use of high-throughput sequencing in clinical practice in past years, cases of a Multilocus Inherited Neoplasia Allele Syndrome (MINAS) were described. Combined P/LP variants have been described for *FLCN*, *VHL*, *FH*, and *SDHB/C* with other genes [25]. For example, a case of MINAS with the development of various types of renal tumors, skin melanoma, breast cancer, and papillary thyroid cancer was characterized by *FLCN* and *BRCA2* pathogenic variants [26]; cases of renal and brain tumors harboring germline pathogenic variants in the *FLCN* and *TP53* genes were described [27].

Average age of patients with P/LP variants was 46.9 versus 33.8 years without. We hypothesize that patients with P/LP variants were in the older age group because the clinical manifestations of BHDS develop with age. Therefore, their referral for testing is more justified. The younger group is characterized by greater clinical heterogeneity and may still include patients with other lung diseases that cause a BHDS-like phenotype.

We have compared the spectrum of the main phenotypic manifestations of BHDS in carriers of P/LP variants in our cohort with the data from other studies (Table 5). The most common clinical manifestation of BHDS in patients with P/LP variants, as in our sample, were pathological changes in the lungs, multiple cysts, and a history of spontaneous pneumothorax. No genotype–phenotype correlations were found, perhaps due to the small number of patients with P/LP variants in our study. Some authors reported association of the *FLCN* LOF variants (frameshift and large deletions) with the risk of pneumothorax compared with point P/LP variants of other types. Thus, deletion of exons 1–3 in the *FLCN* is associated with pneumothorax in eastern China (91% vs. 58%, *p* = 0.047) [22].

Common manifestations of BHDS in Europe, Asia, and America were pulmonary cysts and pneumothorax, while the frequency of RC and fibrofolliculomas varied significantly. The risk of spontaneous pneumothorax in BHDS was 61% as a whole, although it was significantly higher in Asian countries than in Europe: 71 vs. 43%, respectively, according to the results of a meta-analysis [28]. Family history of spontaneous pneumothorax in BHDS serves as an independent prognostic factor for this complication [29]. Based on lung MRI, an algorithm for selecting patients with suspected BHDS for genetic testing had been proposed, which was based on bilateral cysts and their localization in the basal part of the lungs, maximum diameter, and morphological differences in the structure; the clinical sensitivity and specificity of this multivariate model were 95 and 81%, respectively [30].

**Table 5:** Clinical manifestation of BHDS in patients with P/LP variants in the *FLCN*.

Reference	*n*, abs.	Lung Cysts and/or Pneumothorax, %	Renal Tumor, %	Fibrofolliculomas, %	Country
Our study	14	93	21	29	Russia
[11]	35	83	3	9	USA
[31]	26	65	12	16	South Korea
[22]	25	100	20	6	China
[23]	186	66	16	47	Sweden
[32]	101	82	12	58	Denmark

Legend: P/LP—pathogenic/likely pathogenic variants, references are given in square brackets, *n*—absolute number of patients.

Although skin fibrofolliculomas are one of the frequent manifestations of BHDS, they begin to develop mainly in adults aged 30–50 years. Therefore may be reported in a proportion of cases comparable to pulmonary pathology when cumulative risk is assessed by the age of 70, but underestimated in studies devoted to BHDS in young adults [3,32]. It also cannot be ruled out that a few fibrofolliculomas may be underdiagnosed in young patients. This observation was confirmed by a meta-analysis of 204 families with BHDS from various European countries, the USA, Southeast Asia, and Australia: the cumulative risk by the age of 70 in men is 19% for RC, 87% for pulmonary manifestations, and 87% for fibrofolliculomas [33]. As noted above, ChRCC is an informative diagnostic criterion for BHDS, since it occurs in a small percent of sporadic RC cases, but in tens of percent in patients with BHDS. However, other types of renal tumors may also be present in this disease; in particular, the most common type of sporadic kidney tumors, clear cell carcinoma, was identified in 54% of cases with RC in BHDS, while ChRCC and oncocytomas were identified in 32% of cases (a total of 149 patients were examined, 26% of whom were diagnosed with renal tumors) [34]. Bilateral ChRCCs and renal oncocytomas are significantly more informative for the diagnostics of BHDS; families with BHDS manifesting as familial RC in several generations have been described [35,36].

Discussion about clinical criteria for BHDS, which allow a decreased number of patients for molecular genetic diagnostics, has been ongoing for a long time. In 2009, the European BHDS Consortium identified the features required for a diagnosis of BHDS: the presence of one major or two minor criteria. The major criteria are: the presence of at least five fibrofolliculomas (trichodiscomas) in an adult, at least one of which is confirmed histologically, and an identified P/LP variant in the *FLCN*. The minor criteria are: multiple cysts in the lungs in the absence of another known cause, with or without spontaneous pneumothorax; RC before the age of 50 years and/or bilateral/multifocal RC, and/or RC with chromophobe-oncocytic structure; a first-degree relative with BHDS [37]. Currently, the current European ERN GENTURIS recommendations require the presence of at least one criterion from the following: primary spontaneous pneumothorax; multiple bilateral pulmonary cysts (especially in the basal regions) with no other known cause of the disease; bilateral and/or multifocal renal tumors; RC before the age of 50 years or a family history of RC; multiple fibrofolliculomas (trichodiscomas) of the skin; a combination of the above-mentioned features in the proband or a family member, regardless of the family history of the disease [17]. The updated criteria are broader than the previous ones, and this is justified in terms of increasing the sensitivity of BHDS diagnostics. For example, in our work, the pathogenic nonsense variant c.1528G>T (p.Glu510*) was detected in a 26-year-old female patient with infiltrative tuberculosis of the lower lobe of the right lung. A pulmonologist noticed multiple cysts not only in the affected lung tissue on the right, but also in the left lung, and the patient was promptly consulted by a geneticist. She did not meet enough criteria according to the 2009 recommendations, but it was sufficient according to the modern European consensus, which was preferably used in our work. It should be noted that Chinese colleagues, in considering the reduced frequency of cutaneous manifestations of BHDS in East Asia patients versus Caucasians, developed their own recommendations for the diagnosis of patients with BHDS [38].

We would like to note several limitations of our study. First, we examined only the coding regions in the *FLCN* gene in all patients and performed MLPA testing in only 26 patients, which may have resulted in a lack of causative variants in the noncoding region and CNV in the *FLCN* gene, respectively. Second, clinical data were unavailable or available in a limited format for 39 patients, which may have impacted the genotype-phenotype association analysis. Finally, we used the ACMG criteria system for variant classification due to its continued widespread use in diagnostic laboratories, whereas the CanVIG/ACGS criteria are more suitable for diagnostics of hereditary cancer syndromes.

**Figure 3:**
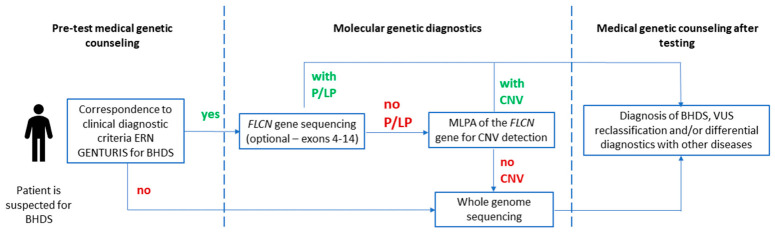
Algorithm for molecular genetic diagnostics of BHDS. Legend: BHDS—Birt–Hogg–Dubé syndrome, MLPA—multiplex ligation-dependent probe amplification, CNV—copy number variation, VUS—variant of uncertain significance.

Clinical diagnostic criteria are often developed to organize and narrow the indications for molecular genetic testing, considering its throughput and laboratory costs. However, WGS is now becoming a routine diagnostic tool. So, the optimal patient pathway for BHDS diagnostics appears to be as follows (Figure 3).

Diagnosis of BHDS using high-throughput genetic analysis methods may be much more relevant than it was 10–20 years ago. Currently, frequency of P/LP variants in the *FLCN* gene according to British biobanks is 1 per 3000–5000 individuals [12]. A similar study of 135,990 exomes in the USA demonstrates this frequency as approximately 1 per 3300 [11], and calculations based on data from the gnomAD database indicate 1 per 3.7–5.7 thousand in Caucasians [39]. WGS allows identification of germline variants in all genes associated with the both BHDS and a BHDS-like phenotypes in the absence of the *FLCN* pathogenic variants [40].

## 4. Materials and Methods

**Samples.** The study included 121 unrelated patients with diagnostic features of BHDS: lung cysts, a history of pneumothorax, cutaneous fibrofolliculomas, and/or chromophobe RC (additionally: oncocytomas, hybrid chromophobe–oncocytic tumors, renal cell carcinoma without specifying the tumor type), as well as 29 of their relatives. The average age of patients at presentation was 32.75 years (range, 1 year to 73 years); 55% were men and 45% were women.

**DNA isolation.** Blood was collected into tubes containing EDTA. Genomic DNA was then isolated from the blood using the ExtractDNA Blood & Cells kit (“Eurogen”, Moscow, Russia) according to the manufacturer’s instructions. The concentration of the extracted DNA was determined fluorimetrically using a Qubit 3.0 instrument and the Qubit™ dsDNA HS (High Sensitivity) Assay Kit (Thermo Fisher Scientific, Waltham, MA, USA).

**PCR and Sanger sequencing.** Protein-coding exons 4–14 of the *FLCN* gene were amplified by polymerase chain reaction (PCR). The reaction mixture consisted of 50–100 ng genomic DNA, 2.5 mM MgCl_2_, 1.5 mM of each dNTP, 2 pmol of forward and reverse primers, 1 unit of thermostable Taq-polymerase, 5 μL of 10× PCR buffer (SibEnzyme, Novosibirsk, Russia), and the volume of the mixture was 25 μL. PCR was performed in a C1000 Touch Thermal Cycler (Bio-Rad Laboratories, Hercules, CA, USA). Primer sequences have been previously published [41]. PCR products were treated with 2 u.a. of E. coli exonuclease I (Fermentas, Vilnius, Lithuania) and 1 u.a. of alkaline phosphatase from calf intestine (SibEnzyme, Novosibirsk, Russia): incubation for 1 h at 37 °C, followed by enzyme inactivation at 85 °C for 15 min. Sanger sequencing was performed using the BigDye^®^ Terminator v3.1 Cycle Sequencing Kit according to the manufacturer’s instructions. Fluorescent products were detected on a 3500 Genetic Analyzer (Thermo Fisher Scientific, Waltham, MA, USA). Sequencing chromatograms were analyzed using Chromas v.2.6.6 software (Technelysium, Brisbane, Australia).

**MLPA for detection of extended deletions of the *FLCN* gene.** Multiplex ligation-dependent probe amplification (MLPA) was used to detect extended deletions of the *FLCN* gene. The SALSA MLPA Probemix P256 FLCN probe kit and other reagents required for hybridization, ligation, and amplification were purchased from MRC Holland (Amsterdam, Netherlands). MLPA was performed according to the manufacturer’s protocols. The MLPA reaction included 50–250 ng of genomic DNA. Fragment analysis was performed on a 3500 capillary genetic analyzer (Thermo Fisher Scientific, Waltham, MA, USA). Chromatogram peak intensity and distribution were analyzed using Coffalyser v.04; heterozygous deletion corresponded to a value of 0.40 < FR < 0.65 (MRC Holland, Amsterdam, Netherlands). The P256 array contains 1–2 probe pairs for each *FLCN* exon 1–14, additional probes for cytosine deletion/insertion in the C8 tract of exon 11 of the *FLCN* gene, and probes for 10 different genomic reference regions not involved in BHDS deletions.

**Clinical significance of genetic variants.** The detected germline genetic variants were designated in accordance with the Human Genome Variation Society (HGVS) nomenclature (https://hgvs-nomenclature.org/, access date 2 November 2025). The pathogenicity of germline variants was determined by the ACMG (American College of Medical Genetics and Genomics) criteria [15] using the Franklin (https://franklin.genoox.com/clinical-db/home (accessed on 5 November 2025)), ClinVar (https://www.ncbi.nlm.nih.gov/clinvar/ (accessed on 5 November 2025)) databases, the own pathogenicity calculator (http://calc.generesearch.ru/ (accessed on 5 November 2025)), and other online tools. The clinical significance of genetic variants was assessed according to Association for Molecular Pathology (AMP) recommendations [42].

**Statistical analysis.** Patient group characteristics include age, sex, lung alterations, renal tumor type, skin lesions, and personal and family anamnesis. Descriptive statistics such as mean and standard error, characteristics of distribution were calculated using Excel (Microsoft Corporation, Redmond, WA, USA) and STATISTICA v.10 (StatSoft, Tulsa, OK, USA). Groups with P/LP vs. “wild type” *FLCN* were compared by age using the nonparametric Mann–Whitney test due to asymmetry in distribution. Patients with pulmonary cysts and/or pneumothorax, renal tumor, and fibrofolliculomas were compared using a two-tailed Fisher’s exact test with a Bonferroni correction. Differences were significant at *p* < 0.05.

## 5. Conclusions

We examined 150 Russian potential BHDS patients and their relatives. P/LP variants in the *FLCN* gene were identified in 25 families. The frequent P/LP variants were localized to exon 11, and six new *FLCN* germline variants were identified. The most common clinical manifestations of BHDS in Russian patients were pulmonary alterations (cysts and spontaneous pneumothorax), while the most informative was a combination of several manifestations of this syndrome, including renal and skin tumors. Currently, the optimal approach is to refer patients who meet the European ERN GENTURIS criteria for diagnosis. Then, the laboratory diagnostics involves sequencing of exons 4–14 of *FLCN* because exons 1–3 are non-coding. If the results are negative, *FLCN* deletions are excluded using MLPA, and, in the absence of CNVs, WGS is performed. This will allow to conduct differential diagnostics with other cancer syndromes and hereditary lung diseases.

If a patient has specific clinical features or a combination specifying hereditary cancer syndrome associated with renal tumor, it makes sense to first perform differential diagnostics instead of WGS. This is especially true since these are often monogenic diseases requiring relatively quick and inexpensive tests (multigene panel NGS or Sanger sequencing). For example, multiple papillary RC can develop due to germline missense variants in the *MET* gene, while clear cell RC can develop in patients with a pathogenic variant in the *BAP1*, *TSC1/2*, or *VHL* gene (especially if RC is combined in a personal and/or family history, including clinical signs of von Hippel–Lindau syndrome such as central nervous system hemangioblastoma, pheochromocytoma, and pancreatic/renal cysts). Given the emerging evidence of a higher frequency of P/LP variants of the *FLCN* in Caucasians, effective diagnosis of BHDS appears particularly relevant. The increasing availability of WGS may bring this method to the forefront of routine testing in patients suspected for BHDS.

## Figures and Tables

**Table 1:** Point *FLCN* gene variants in patients with BHDS.

Number of Cases	Germline Variant	*FLCN* Exon	ACMG Criteria (Points)	Pathogenicity (ACMG)	CanVIG	Variant Type
1	c.214dup (p.Ser72Lysfs*28)	4	PVS1(8) + PM2(2)	P	P	frameshift
1	c.247G>A (p.Glu83Lys)	4	PM2(2) + BP6(−1)	VUS	LB	missense
1	c.249+5G>C	-	PM2(2) + PP3(2) +PP5(1)	VUS	LP	splicing
1	c.328C>T (p.Gln110*)	5	PVS1(8) + PM2(1)	LP	P	nonsense
1	c.346C>A (p.Gln116Lys)	5	PM(2) + BP1(−1) + BP6(−1)	VUS	LB	missense
1	c.346dupC (p.Gln116Profs*17)	5	PVS1(8) + PM2(1)	LP	P	frameshift
1	c.390C>G (p.Ser130Arg)	5	PP3(2) + PM2(1) + BP1(−1)	VUS	VUS	missense
1	c.476_479dup (p.Asp160Glufs*41)	6	PVS1(8) + PM2(2) + PP5(1)	P	P	frameshift
1	c.490del (p.Arg164Glyfs*13)	6	PVS1(8) + PM2(2) + PS4(2)	P	P	frameshift
1	c.494del (p.Gly165Alafs*12)	6	PVS1(8) + PM2(1)	LP	P	frameshift
1	c.499C>T (p.Gln167*)	6	PVS1(8) + PM2(2) + PS2(1)	P	P	nonsense
1	c.715C>T (p.Arg239Cys)	7	PS3(1) + PP3(1) + BP1(−1) + BP6(−1)	VUS	LB	missense
1	c.931_941del (p.Pro311Argfs*75)	9	PVS1(8) + PM2(2)	P	P	frameshift
2	c.970C>T (p.Gln324*)	9	PVS1(8) + PM2(2) + PP5(1)	P	P	nonsense
1	c.1015C>T (p.Gln339*)	9	PVS1(8) + PM2(2) + PS4(2)	P	P	nonsense
1	c.1067T>C (p.Leu356Pro)	10	PM2(2) + PP3(2)	VUS	VUS	missense
5	c.1285del (p.His429Thrfs*39)	11	PVS1(8) + PM2(2) + PS4(2)	P	P	frameshift
1	c.1293_1300+19del	11	PVS1(8) + PM2(2) + PS4(1)	P	P	splicing
4	c.1300G>C (p.Glu434Gln)	11	PS4(2) + PM2(2) + PM5(2) + PP3(1)	LP	LP	missense
1	c.1390G>T (p.Glu464*)	12	PVS1(8) + PM2(2) + PS4(2)	P	P	nonsense
1	c.1522_1524del (p.Lys508del)	13	PS4(4) + PS3(1) + PM1(2) + PM2(2) + PM4(2) + PM5(1)	P	P	inframe deletion
1	c.1528G>T (p.Glu510*)	13	PVS1(8) + PM2(2)	P	P	nonsense
1	c.1658G>A (p.Trp553*)	14	PVS1(8) + PM2(1)	LP	P	nonsense
1	c.1538+5G>C	-	PM2(2) + PP3(2)	VUS		splicing

Legend: P—pathogenic, LP—likely pathogenic, VUS—variant of uncertain significance, LB—likely benign, PVS—power very strong, PS—power strong, PM—power moderate, PP—pathogenic supporting, and BP—benign supporting (criteria); ACMG—American College of Medical Genetics and Genomics, CanVIG—Cancer Variant Interpretation Group UK.

**Table 2:** Characteristics of the VUSes.

Variant	gnomAD	RuExAc	AlpaMissense	PolyPhen2	REVEL	ClinVar	Varsome	Franklin
	Population Frequency Databases		In Silico Predictors			Pathogenicity Classificators		
**Missense VUSes**								
c.247G>A (p.Glu83Lys)	0.00001780	no	0.775 uncertain	0.014 benign	0.546 likely disease causing	LB/VUS	LB	VUS
c.346C>A (p.Gln116Lys)	0.00007034	0.0010309	0.202 benign	0.636 possible damaging	0.509 likely disease causing	LB/VUS	LB	VUS
c.390C>G (p.Ser130Arg)	no	no	1.000 strong pathogenic	1.000 probably damaging	no	no	VUS	VUS
c.715C>T (p.Arg239Cys)	0.0005593	no	0.988 pathogenic moderate	0.999 probably damaging	0.914 likely disease causing	B/LB/VUS	LB	VUS
c.1067T>C (p.Leu356Pro)	no	no	0.983 pathogenic moderate	0.958 probably damaging	0.841 likely disease causing	VUS	VUS	VUS
**Splicing VUSes**								
			**dbscSNV**	**SpliceAI**	**MaxEntScan**			
c.249+5G>C	no	no	no	0.88 donor loss	2.3064 uncertain	VUS/P	VUS	LP
c.1538+5G>C	no	no	0.9999 pathogenic strong	0.82 donor loss	4.6962 pathogenic supporting	VUS	VUS	VUS

Legend: B—benign, LB—likely benign, VUS—variant of uncertain significance, LP—likely pathogenic, P—pathogenic.

**Table 3:** Patient’s phenotypes with clinically BHDS.

Pathological Feature	with P/LP, *n* = 14	No P/LP, *n* = 68	*p* **
Pulmonary cysts and/or, pneumothorax	13 (93%)	51 (75%)	0.284
Renal tumor	3 (21%)	9 (13%)	0.422
Fibrofolliculomas	**4 (29%)**	**4 (6%)**	**0.022**
Multiple (combined) alterations *	**14 (100%)**	**37 (54%)**	**0.001**

Legend: * recurrent pneumothorax and/or bilateral (multifocal) renal tumors, ** probability of the null hypothesis; P/LP—pathogenic/likely pathogenic variants.

**Table 4:** Patients’ relatives tested for identified variant.

Purpose of Analysis	with P/LP, *n* = 7	No P/LP, *n* = 22
VUS reclassification	3	6
Validation in children	3	5
Validation in other 1st and 2nd degree relatives	1	11

Legend: P/LP—pathogenic/likely pathogenic variants, VUS—variant of uncertain significance.

## Data Availability

The original contributions presented in this study are included in the article. Further inquiries can be directed to the corresponding author.

## Abbreviations

The following abbreviations are used in this manuscript:

ACMG	American College of Medical Genetics and Genomics
AMP	Association of Molecular Pathology
BHDS	Birt-Hogg-Dubé Syndrome
ChRCC	Chromophobe Renal Cell Carcinoma
CNV	Copy Number Variation
DNA	Deoxyribonucleic Acid
dNTP	Deoxyribonucleic Triphosphate
EDTA	Sodium EthyleneDiamine TetraAcetate
HGMD	Human Genome Mutation Database
HGVS	Human Genome Variation Society
LOF	Loss Of Function
MINAS	Multilocus Inherited Neoplasia Allele Syndrome
MLPA	Multiplex Ligation-dependent Probe Amplification
MRI	Magnetic Resonance Imaging
NCCN	National Comprehensive Cancer Network
OMIM	Online Mendelian Inheritance in Man
PCR	Polymerase Chain Reaction
P/LP	Pathogenic/Likely Pathogenic variant
RC	Renal Cancer
VUS	Variant of Uncertain Significance
USA	United States of America
WGS	Whole-Genome Sequencing
